# 3D Bioprinting of Vascularized Tissues for *in vitro* and *in vivo* Applications

**DOI:** 10.3389/fbioe.2021.664188

**Published:** 2021-05-13

**Authors:** Earnest P. Chen, Zeren Toksoy, Bruce A. Davis, John P. Geibel

**Affiliations:** ^1^Department of Surgery, School of Medicine, Yale University, New Haven, CT, United States; ^2^Yale College, Yale University, New Haven, CT, United States; ^3^Department of Cellular and Molecular Physiology, School of Medicine, Yale University, New Haven, CT, United States

**Keywords:** 3D bioprinting, additive manufacturing, tissue engineering, bioprinting, vasculature

## Abstract

With a limited supply of organ donors and available organs for transplantation, the aim of tissue engineering with three-dimensional (3D) bioprinting technology is to construct fully functional and viable tissue and organ replacements for various clinical applications. 3D bioprinting allows for the customization of complex tissue architecture with numerous combinations of materials and printing methods to build different tissue types, and eventually fully functional replacement organs. The main challenge of maintaining 3D printed tissue viability is the inclusion of complex vascular networks for nutrient transport and waste disposal. Rapid development and discoveries in recent years have taken huge strides toward perfecting the incorporation of vascular networks in 3D printed tissue and organs. In this review, we will discuss the latest advancements in fabricating vascularized tissue and organs including novel strategies and materials, and their applications. Our discussion will begin with the exploration of printing vasculature, progress through the current statuses of bioprinting tissue/organoids from bone to muscles to organs, and conclude with relevant applications for *in vitro* models and drug testing. We will also explore and discuss the current limitations of vascularized tissue engineering and some of the promising future directions this technology may bring.

## Introduction

**Figure 1:**
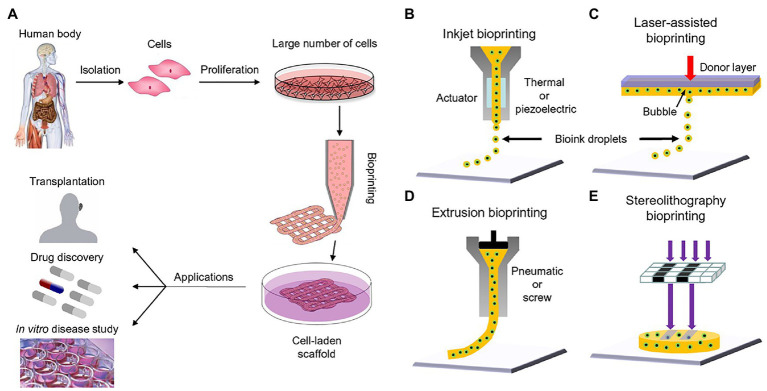
Bioprinting process and techniques. **(A)** The typical workflow of bioprinting starts with the choosing the right type of cells, then culturing cells and preparing the bio-ink, printing the desired cell-laden scaffold, and finally used for transplantation, drug testing, or *in vitro* studies. **(B)** Inkjet bioprinters produce small droplets of hydrogel and cells in a sequential manner to construct tissues. **(C)** Laser-assisted bioprinters focuses a light source onto a donor layer (top) which propels the cells onto the print (arrow indicates direction of laser source). **(D)** Extrusion-based bioprinting produces a continuous supply of hydrogel and cells. **(E)** Stereolithography bioprinting uses digital light sources to selectively crosslink bio-inks layer by layer (arrows indicate direction of projected light). Reprinted with permission from Biotechnol. Adv. (Mandrycky et al., 2016), c 2021.

Not only are organ donors a limited resource, the functional efficacy of donated organs as well as risk of disease and/or infection (a prominent issue during the COVID-19 pandemic) from deceased individuals greatly limit the number of available tissues for transplantation (Neuberger and Callaghan, 2020; Shah et al., 2020). Living donors, although providing another small pool of organs or partial organs, still do not keep up with the current demand (Lee et al., 2019c). Thus, ongoing active research has pursued alternative methods of obtaining functional tissues for implantation (Cui et al., 2017). In response to the demand outpacing the availability and with our expanding knowledge of cellular biology, the progress of employing tissue engineering research and its medical applications has come a long way in the past decades (Zhang et al., 2018). Specifically, the incorporation of three-dimensional (3D) bioprinting technology in the field of regenerative medicine has created new possibilities for patient-specific tissue regeneration therapy (Figure 1A; Mandrycky et al., 2016; Kačarević et al., 2018).

3D bioprinting or additive manufacture allows for the precise layer-by-layer construction of different complex cell models and tissue types with high precision, repeatability, and reproducibility (Li et al., 2016; Bishop et al., 2017; Kačarević et al., 2018). 3D bioprinting technology has the ability to control the external shape and the internal geometry, spatial distributions, and cellular orientation of generated tissues to recapitulate the structure and function of their native counterparts (Lee and Yeong, 2016; Zhu et al., 2016; Bishop et al., 2017; Murphy et al., 2020). Numerous printing methods have been explored and implemented for the fabrication of various tissue architectures including: extrusion bioprinting, inkjet bioprinting, laser assisted bioprinting, dual head printing, and light-mediated stereolithography (Figures 1B–E; Mandrycky et al., 2016; Derakhshanfar et al., 2018). The choice of “bioinks” and/or scaffolds for 3D bioprinting also determines tissue integrity, interconnectivity of different components within the organ, and temporal release of growth factors and bioactive substances for proper tissue development or repair (Zhu et al., 2016; Zhang et al., 2017; Gungor-Ozkerim et al., 2018). By incorporating developmental biology, cell biology, regenerative medicine, and bioengineering and specifically tissue engineering, 3D bioprinting provides a platform for artificially constructing accurate tissue mimics for patient implantation and drug testing (Bishop et al., 2017). Figure 1A shows a summary illustration on how to harvest cells for bioprinting and also what type of printers is commonly employed at this time.

In the following sections of this review, we will examine the present state of the published literature and focus also on recent advances, accomplishments, and new directions in this field. This review will address both *in vitro* and *in vivo* applications of the technology to date, and will, in subsequent sections, examine applications of bioprinting for organoid development that is being applied for drug development. We have included limitations of each technique that has been used and suggest some recommendations for future development of 3D bioprinted biological material. Studies involving *in vitro* applications will be in bold italics and for *in vivo* applications will be listed with underlined bold italics to aid the reader in identifying where the studies were performed and to act as the indices for the review.

We will first discuss the current methods for engineering vascular networks and blood vessels in fabricated tissue. This section will be followed by an examination of some of the exciting current advances in the most researched areas of bioprinting: bone, muscle, cardiac, liver, and skin and discuss attempts to implant these engineered tissues *in vivo*. We have chosen this order so that we begin with the vasculature and then move our discussion of recent attempts to make bone replacements and the need for vascular conduits to provide nutrients and to remove waste from the printed bones. Since bioprinted bone has now reached the level of *in vivo* implantation, the ability for this material to incorporate with the native bone and surrounding tissue will be addressed. In the subsequent sections, we will address attempts to design bioprinted replacement organs and the issues of designing and printing a complex multicellular tissue that also requires connection to a blood supply for nutrient delivery and waste removal. Lastly, we will discuss the current advances in constructing tissue and cellular mimics (bioengineered organoids) for drug testing and disease modeling for both physiology and pathophysiology.

## Vascularization of Tissues

Tissues and organs in our body are highly vascularized to allow for gas exchange (oxygen-CO_2_) along with: salt, water, and nutrient diffusion through the tissue and waste disposal (Tomasina et al., 2019). Capillary distance within tissues is 60–300 μm, and survival rates of cells populating areas above this diffusional distance limit dramatically declines (Krogh, 1919; Kety, 1951; 16; Bainbridge, 2013). With this caveat, incorporation of vasculature into bioprinted products is critical for the sustained growth and survival of the tissue, and to allow for maintenance of normal physiological viability (Tomasina et al., 2019).

There are currently two main strategies for blood vessel incorporation in bioprinted material. The first of these relies on the controlled release of angiogenic factors that help induce blood vessel growth in 3D printed tissues (Inglis et al., 2016; Park et al., 2019). This technique has been employed in ongoing active research where the primary goal is patient implantation of the printed material (Inglis et al., 2016; Datta et al., 2017). The second strategy involves the direct printing of vascular scaffolds (Datta et al., 2017). The target tissue cells will then be directly printed on or around the scaffold to build a vascularized organoid construct (Datta et al., 2017). Preprinted vascular networks and the direct printing of blood vessels are frequently found in the development of *in vitro* tissue samples for drug testing and metabolic assays (Datta et al., 2017).

In the next section of the review, we will discuss current developments in the preparation of vascular conduits which are essential to allow for print survival in organs and tissues. This section will be followed by the examination of deployment of these conduits in aiding the development of: functional bone printing, muscle printing, heart printing, liver printing, and finally a discussion of skin bioprinting.

### Preparation of Vascular Conduits

**Figure 2:**
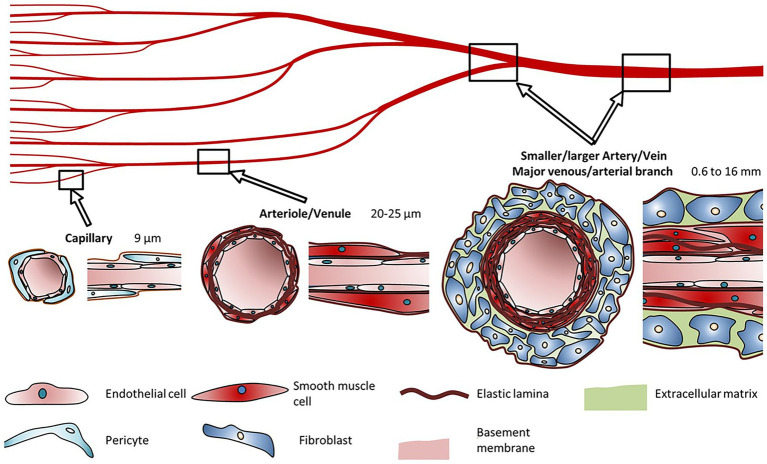
Blood vessel diameters, cell types, and compositions. Capillaries are typically made up of pericytes surrounding an extracellular lining. Arterioles and venules are surrounded by smooth muscle cells. Arteries and veins consist of fibroblasts surrounding a layer of smooth muscle cells. Reprinted with permission from Nat. Sci. Rep. (Schöneberg et al., 2018), c 2021.

A fundamental necessity for the development of viable bioprinted artificial tissue(s) is a vasculature network that will provide a pathway for nutrient absorption and excrement removal to maintain normal pathophysiological function. When examining the printed tissue’s physiological needs, we see that the limit of diffusion for O_2_ in tissue is generally accepted to be limited to 100–200 μm (Krogh, 1919; Carmeliet and Jain, 2000; Jain et al., 2005). This printed vascular network is essential for the delivery of nutrients and oxygen along with the removal of waste products and CO_2_ (Jafarkhani et al., 2019). The vasculature in the human body ranges from very large (mm size) to microscopic (μm; Figure 2; Schöneberg et al., 2018; Tomasina et al., 2019). Ideally the goal remains to produce vessels that resemble the endogenous system in terms of biocompatibility, physiological flow rates, and ability to withstand systemic pressure changes.

Several methods have been explored and evaluated for bioprinting of blood vessels. These methods generally attempt to mimic a native blood vessel in that they include endothelial cells of the intima, and smooth muscle cells of the media and a structural layer mimicking the adventitia (Mazurek et al., 2017). The first biosynthetic blood vessel was created by Weinberg and Bell in 1986 (Weinberg and Bell, 1986). Weinberg’s vessel consisted of smooth muscle cells grown in a collagen gel in an annular mold. This was then covered with a Dacron mesh followed by an outer layer of fibroblasts. The tubular construct was removed from the mandrel and an endothelial layer of cells was added to the inner layer. A limiting factor was the necessity of culturing sufficient autologous endothelial cells for the adventitia (Weinberg and Bell, 1986). The burst pressure was also found to be insufficient for use in high pressure vessels. This initial advancement, however, gave rise to numerous other studies using other synthetic materials and cultured cells (Chlupác et al., 2009). The stability of the endothelial layer and synthetic biocompatibility has been of some concern for the continued long-term survival of the printed vessel. The first entirely biological vessel construct was completed in 1998 by L'Heureux who grew a sheet of smooth muscle cells which was then wrapped around a tubular support (L’Heureux et al., 1998). A sheet of fibroblasts was grown and then wrapped over the smooth muscle cells. Finally, the smooth muscle/fibroblast tube was removed from the mandrel and endothelial cells were grown on the inner wall of the tube (L’Heureux et al., 1998). The cell culture involved in this multilayer approach took around 8–12 weeks to complete the process. This long timeframe remains a limiting factor for mass production of replacement tissues and also for situations necessitating a rapid deployment in critical applications for patients. This time frame would become even longer if the cells used were harvested directly from the patient that requires a replacement vessel. These initial studies also needed to examine inflammation associated with a multilayer print and the innate immune system that could recognize this as a foreign body.

The next step in the history of tissue engineered vascular grafts (TEVG), and other physiological bioprinting applications was the use of absorbable or degradable support scaffolds (Matsuzaki et al., 2019). Biopolymers, such as polyglycolic acid (PGA), have been used since the early 1970s when they were developed for use as absorbable sutures (Frazza and Schmitt, 1971; Miln et al., 1972; Reed and Gilding, 1981). Foam polymers such as poly(L-lactic) acid (PLA) and poly(DL-lactic-co-glycolic) acid (PLGA) were examined for use as scaffolds for cell transplantation (Mikos et al., 1993; Wald et al., 1993). Niklason and Langer (1997) grew smooth muscle cell on tubular PGA mesh. A periodic stretch of the SMC-scaffold construct during cell culture helped maintain the smooth muscle physiological characteristics. However, these vessels were unable to withstand normal arterial pressures (Niklason and Langer, 1997). Mechanical stretching during culture was shown to improve the strength of SMC grown on a collagen-gel scaffold (Seliktar et al., 2000). Improved culturing conditions resulted in a successful implantation of an engineered vessel *in vivo* and were accomplished in 1999. A prolonged culture period (8 weeks) is still something to be improved upon to allow for urgent use in trauma situations. Luo et al. (2020) has recently successfully demonstrated the use of Induced pluripotent stem cells (ihPSC) for TEVG using a PGA scaffold.

#### Limitations and Future Suggestions

As discussed in the previous section, the main limitations of the technologies deployed to date focus around the time to prepare and generate sufficient quantities of cells that will be used in the generation of the print. Also, in the early attempts in the deployment of 3D bioprinting for this application, it was shown that even after the generation of the print, there is a relatively long time of conditioning the print (in some cases 2–3 months) prior to attempting to deploy the engineered replacement. During the development of these prints, issues associated with longevity and also burst pressures were researched *in vitro*. With the present technologies, it makes having a patient specific application prepared for emergency use a nonstarter. Some potential suggestions are to create a series of conduits that are generated from stem cells that are devoid of antigen activity that could become universal implants for emergency situations. To create this technology, there is still going to be a time lag for creating the print and also for its storage. This also does not address if it will be possible to have only large conduits or if small and branched conduits can also be created that will have sufficient viability while being stored. Another possible future development could be the use of different printer modalities that may extrude printed material at higher rates, at low or high density, and print using less support material or potentially lower cell densities.

In the next section of the review, we will review the presently deployed printing technologies.

### Deployed Printing Technologies

Based on previous works and studies currently underway, there are three methods employed for bioprinting vasculature: extrusion, inkjet (droplet), and laser based (Kačarević et al., 2018; see Figure 1). In extrusion based bioprinting, the cells (generally endothelial and or smooth muscle cells) are suspended in a hydrogel consisting of alginate, fibrin, PEG, and gelatin which serve as a scaffold (Axpe and Oyen, 2016). After extrusion, the cell-hydrogel mix (bioink) undergoes physical or chemical crosslinking which occurs allowing the gel to maintain the desired printed shape (Skardal et al., 2010; Gungor-Ozkerim et al., 2018; Maina et al., 2020). Tubular constructs can be printed by extrusion of the hydro-gel in a vertical network of hollow tubules (Li et al., 2009), printing around a solid mandrel (Maina et al., 2020) or coaxial printing (Zhang et al., 2013a; Datta et al., 2017). The method described here is referred to as “direct extrusion.” The printed construct is directly extruded into the final form in this case a tubule. Alternatively, indirect extrusion can be accomplished by printing the tubular shape using a sacrificial hydrogel which contains no cells and is printed into or on a structural hydrogel (Hinton et al., 2015; Datta et al., 2017). The sacrificial hydrogel is removed and the remaining network is perfused with media containing vascular cells (Zhang et al., 2013b; Datta et al., 2017; Maina et al., 2020).

The droplet (inkjet) based method of bioprinting utilizing acoustic (piezoelectric), thermal, and/or electrostatic technologies allowing for intricate patterns of cell deposition (Cui et al., 2012; Gudapati et al., 2016; Datta et al., 2017). As described above, inkjet bioprinting uses cells suspended in a liquid hydrogel which undergoes gelation after being deposited on the target. Sanjana and Fuller (2004) described this process using rat hippocampal neuron cells in a poly-D-lysine hydrogel. This technique has several advantages such as a relatively low-cost platform, improved cell viability, and precision deposition (Sanjana and Fuller, 2004; Ringeisen et al., 2006; Cui et al., 2012).

Another novel exciting method of bioprinting is laser based bioprinting. This technique allows for precise and high-resolution printing using a laser as a source of energy (Michael et al., 2013; Zhu et al., 2016; Papaioannou et al., 2019). This technique can be used to generate scaffolds onto which cells can be built (digital light processing, DPL; Zhu et al., 2016) or alternatively, it can be used to print cells onto a surface (Murphy and Atala, 2014; Zhu et al., 2016). This has been labeled laser induced forward transfer (LIFT; Yusupov et al., 2020). The cell laden bioink is placed on the surface of a plate or slide. The laser is focused on the side opposite to the bioink creating a microbubble that ejects a tiny droplet of cells-bioink (Koch et al., 2010, 2012; Schiele et al., 2010). LIFT is capable of producing precise and high-resolution printing of cells while avoiding many issues associated with alternative printing methods such as clogging and contamination thanks to its nozzle-free and non-contact nature.

#### Limitations and Future Suggestions

As reviewed above, each of the present technologies has limitations preventing the selection of a single technology that could be used universally for all applications. Clearly, the use of laser assisted bioprinting seems a potential means to deliver a focused density of cells in a pattern at a specific location; however, there remains the issues of thermal damage during the heating process and that this could become an important issue if one attempts to print small complex tissues where the thermal gradient could be transferred to the adjacent cells. With classical extrusion printing, there is a large deal of preparation of the bio-ink that will be used to transfer the cells. Also, the more complex the tissue being printed, the more complex the ink which will require more extrusion print heads to maintain location and orientation during the printing process.

Future suggestions would be centered on a hybrid printer that could have precision of laser-based spray printing as droplets, or even streams of cellular material. If this is coupled to a support gel that the cells are in during this spray process, we may alleviate the potential thermal issues while having the precise deposition of material to create the finished complex print.

In the next section of the review, we will examine the present state of creating *vascularized bone tissues* where investigators have looked at various bone substitutes and are now also looking at providing these printed structures with a vascular supply for nutrient and waste removal.

### Vascularized Bone Tissues

**Figure 3:**
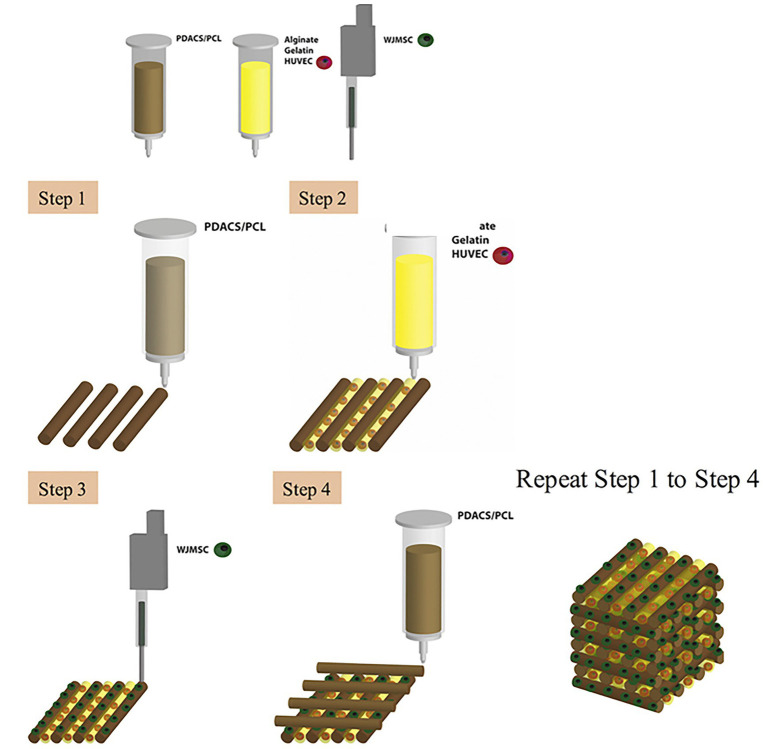
Schematic diagram of the bioprinting process. First, a framework was fabricated with polydopamine-modified calcium silicate (PDACS)/poly-caprolactone (PCL) composite to support the entire mechanical stability. Second, the alginate/gelatin hydrogels encapsulated human umbilical vein endothelial cells (HUVEC) were dispensed into the pores. The Wharton’s jelly mesenchymal cells (WJMSC) were printed on the PDASC/PCL scaffold with the piezoelectric needle. The sequential dispensing of PDACS/PCL composite, hydrogel, and cells was repeated and stacked to build the three-dimensional (3D) scaffold (10 layers). Reprinted with permission from Mater. Sci. Eng. C Mater. Biol. Appl. (Chen et al., 2018), c 2021.

Bone tissue has the strongest mechanical properties within the body (Zhang et al., 2018). The challenge to develop viable bioprinted bone is to incorporate vascular networks without compromising the structural strength/integrity of 3D printed bone when compared to endogenous bone. A great deal of research has been devoted to developing bone tissue that supports the vasculature necessary in biological systems (Chen et al., 2018). Traditionally, biomaterials such as calcium phosphate have been used to substitute missing bone (Chen et al., 2019). Chen et al. (2018) reported the use of calcium silicate (CS) instead of calcium phosphate for the 3D printing of scaffolds for bone as CS has shown to produce calcified bone-like apatite layers that supersede that of calcium phosphate. They constructed a polydopamine-modified calcium silicate (PDACS)/poly-caprolactone (PCL) scaffold in which Wharton’s jelly mesenchymal cells (WJMSCs) and human umbilical vein endothelial cells (HUVECs) used in a combinatory hydrogel that was then incorporated into a single product (Figure 3; Chen et al., 2018). Results from these studies showed that the addition of the PDACS/PCL scaffold into the WJMSCs/HUVECs hydrogel led to higher levels of bone formation proteins and angiogenic biomarkers, suggesting that this approach may be effective for building deep bone structures with complex vascular networks (Chen et al., 2018).

**Figure 4:**
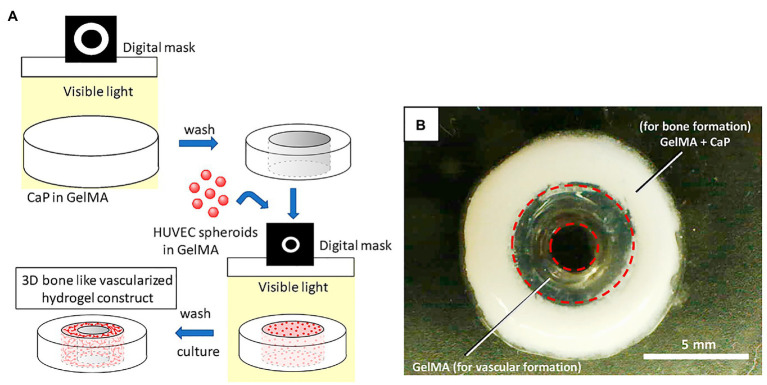
Schematic illustration for 3D hydrogel construct. **(A)** Schematic illustration of fabrication process for 3D hydrogel construct. **(B)** A photograph of 3D hydrogel constructs for vascular and bone formation. Reprinted with permission from Int. J. Mol. Sci. (Anada et al., 2019), c 2021.

**Figure 5:**
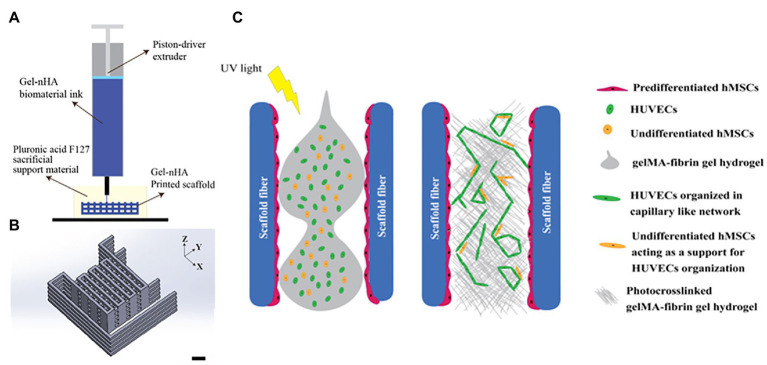
Schematic of the bioplotting technique. **(A)** Schematic of the bioplotting technique used to print wood-pile scaffolds with vertical and lateral pores; a sacrificial support material provides a support to the biomaterial ink during the printing avoiding the collapse of the scaffold. **(B)** Wood-pile scaffold CAD model designed. **(C)** Schematic of the process used to add HUVECs to the bone construct, filling the whole interconnected pore network within the scaffolds. Reprinted with permission from Biofabrication (Chiesa et al., 2020), c 2021.

Different parts of the bone exhibit unique structures and cell composition (Anada et al., 2019). Anada et al. (2019) developed a two-step digital light processing technique that included the use of HUVEC spheroids and octacalcium phosphate (OCP) for printing the complex bone structures (Figure 4). This pattern of *HUVEC spheroids* embedded in gelatin methacrylate (GelMA) in a dual ring structure was designed to mimic the bone marrow space. Results suggest that GelMA concentration modulates the extent of capillary-like structures that originate from HUVEC spheroids, and that 3D bioprinted bone constructs with biomimetic dual ring structures can be potentially used to engineer vascularized bone tissue (Anada et al., 2019). Meanwhile, Cidonio et al. (2019a) utilized a synthetic nanoclay, laponite (LPN) along with GelMA as their bioink for bone tissue construction. In this study, they incorporated vascular endothelial growth factor (VEGF) in the bioink to induce increased angiogenesis after implantation. Results confirm that *VEGF-loaded LPN-GelMA* constructs in vascular chick embryos demonstrated higher blood vessel penetration than previously used GelMA-VEGF scaffolds (Cidonio et al., 2019b). Recently, Chiesa et al. (2020) reports the *in vitro* construction of bone tissue by first seeding a gelatin-nanohydroxyapatite (gel-nHA) scaffold with hMSCs for 2 weeks, and then included HUVECs in the macropores of the scaffold to induce angiogenesis (Figure 5). A bone model with a robust vascular network was realized in 4 weeks’ time with fully developed vascularization in just 2 weeks. Although VEGF doped bioink can improve vascularization, the question that remains is what occurs when you have an increased systemic exposure to VEGF that has been shown to be carcinogenic (Verheul and Pinedo, 2000; French and Frazier, 2011).

**Figure 6:**
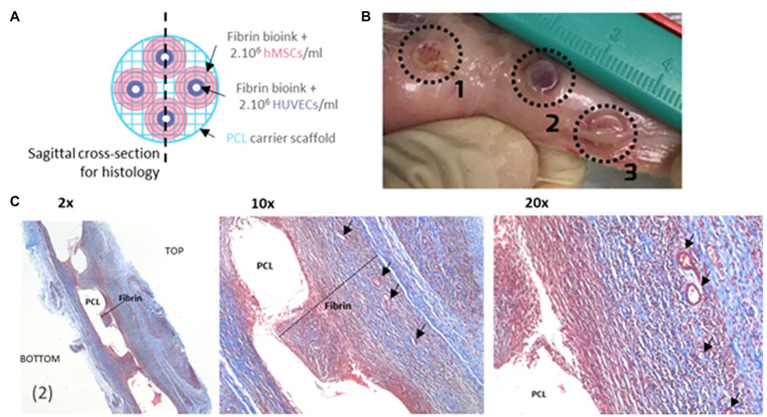
Process of making a 3D bioprinting (3DP) scaffold. **(A)** Schematic of a 3DP scaffold. Osteon-like fibrin hydrogels are co-printed with PCL for support. **(B)** Images of samples after 14 days implantation *in vivo*. **(C)** Micrographs of embedded, sectioned, and stained samples using Masson Trichrome at day 14 after implantation. Collagen is stained blue, cell nuclei are stained dark purple, and fibrin is stained pink. Black arrows indicate blood vessels. Reprinted with permission from Biofabrication (Piard et al., 2019), c 2021.

The reduced cellular density and high tensile strength of bone and its architecture of blood vessel networks stems from the efficient organization of osteocytes. Piard et al. (2019) explored the improvement of bioprinting scaffolds to assist controlled angiogenesis in bone constructs while maintaining the structural integrity. They used extrusion-based 3D bioprinting (3DP) to build a biphasic osteon-like scaffold that contains two separate osteogenic and vasculogenic populations of cells encased in fibrin bioink (Figures 6A,B; Piard et al., 2019). *HUVECs* with human mesenchymal stem cell *(hMSC) laden hydrogels* printed in osten-like patterns *in vitro* induced a significant expression of angiogenic markers and an increase in blood vessel per tissue volume density (Figure 6C; Piard et al., 2019). These results demonstrate that proper scaffold design and cell placement during 3D bioprinting is essential for neovascularization (Piard et al., 2019).

**Figure 7:**
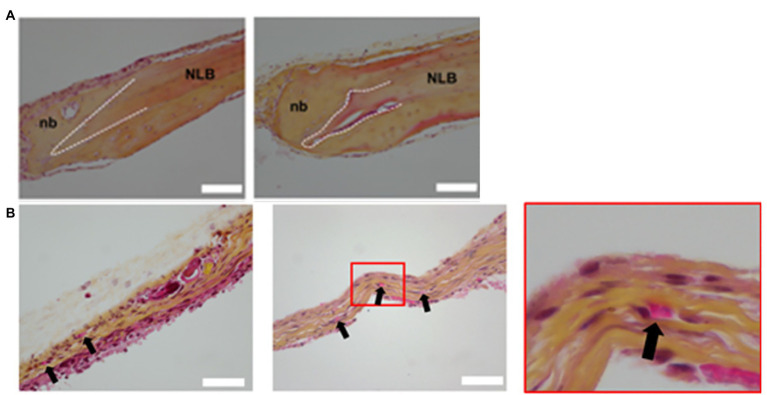
Histology of calvaria. **(A)** Histological examination of decalcified calvaria defects stained with HES staining. The left and right columns show histological images of regeneration area 1 and 2 months post-printing, respectively. NLB, native lamellar bone; nb, neoformed bone. White dash lines show the border of the calvaria defect. **(B)** Histological examination of decalcified calvaria defects stained with hematoxylin-eosin-saffron staining to assess vascularization. The left column shows histological images of regeneration areas 1 month post-printing. The middle and right columns, respectively, display histological images of regeneration areas 2 months post-printing and the magnification of the red squared areas. Black arrows indicate blood vessels. Reprinted with permission from Biofabrication (Kérourédan et al., 2019b), c 2021.

With the maturation of 3D bioprinting technology, the focus now transitions to the incorporation of fabricated tissues into living organisms. Kérourédan et al. (2019b) used laser-assisted bioprinting (L’Heureux et al., 1998) *to construct bone tissue* in mice with calvarial bone defects *in situ*. LAB allowed the precise bioprinting with cell-level resolution of various patterns of endothelial cells, mesenchymal stem cells, collagen, and VEGF into the bone defect (Kérourédan et al., 2019a). Their results demonstrated that the *LAB technique* and the incorporation of VEGF were safe and highly controlled under these conditions (Kérourédan et al., 2019b). The endothelial cells gave rise to organized microvascular networks of significance at 2 months suggesting that *in vivo* bioprinting with LAB is an invaluable tool for bone tissue pre-vascularization not only *in vitro*, but *in situ* (Figure 7; Kérourédan et al., 2019b). Rukavina et al. (2020) constructed pre-vascularized bone tissue with adipose-derived mesenchymal stem cells (ASCs) and HUVECs. The construct was then implanted subcutaneously in immunodeficient mice (Rukavina et al., 2020). Not only did new micro-vessels start to develop, the newly formed vessels were stabilized by mouse pericytes, indicating that the pre-vascularized artificial bone tissue can lead to normal bone and vascular development after implantation (Rukavina et al., 2020).

#### Limitations and Future Suggestions

Although there has been a great deal of progress in developing a variety of bone replacements for implantation to correct defects due to disease or injury the remaining open question is to attach or adapt a blood supply so that the implant not only remains viable but will incorporate into the surrounding bone tissue. Efforts have focused on applying growth factors, specifically VEGF, to try and stimulate blood vessel formation on the surface of the print. In some of the cases discussed above, this has been moderately successful, but it still leaves questions as to what will happen systemically if the VEGF escapes into the endogenous blood supply. Applications of any vascular growth agent, and particularly VEGF, there is a distinct possibility that unwanted cell proliferentiaon can occur outside the area of interest. This could be particularly complicating if the bone replacement was due to cancer. The likelihood of additional tumorogenisi occurring locally or remotely is increased.

### Vascularized Muscle Tissue

**Figure 8:**
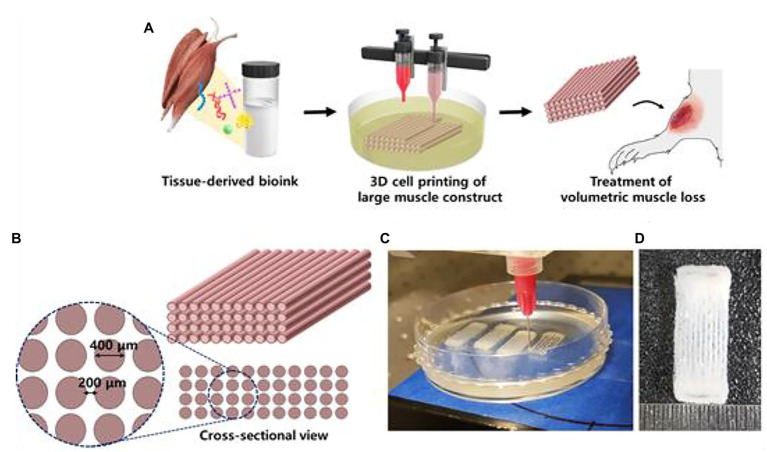
3D cell printing of skeletal muscle construct. **(A)** Schematic illustration of the decellularized extracellular matrix (dECM) bioink preparation, muscle construct fabrication, and volumetric muscle loss (VML) treatment. **(B)** Design of muscle construct. **(C)** 3D cell printing of muscle construct using a granule-based reservoir system. **(D)** 3D cell printed muscle construct. Reprinted with permission from Biomaterials (Choi et al., 2019), c 2021.

Skeletal muscle tissue is made up of a heterogeneous composition of highly differentiated and sophisticated muscle fibers and motor neurons (Kang et al., 2020). Muscle fibers are often damaged by degenerative diseases, traumatic injuries, and tumor ablation, which lead to muscle fiber atrophy (Kang et al., 2020). Although skeletal muscle has high regenerative capacities, large volumes of muscle cannot be recovered without interventional support (Kang et al., 2020). Proper muscle structure and function requires viable blood vessel networks (Kim et al., 2018). Current muscle constructs have been tested *in vivo* on rodent models (Kim and Kim, 2018). Kim et al. (2018) 3D *printed implantable skeletal muscle* tissues that present highly organized multi-layered muscle bundles that resemble biological myofibers using human primary muscle progenitor cells (hMPCs). *In vivo* implantation of fabricated muscle tissue in rodent models with tibialis anterior muscle defects reveal that the muscle constructs regain 82% of lost muscular function in 8 weeks. Histological analyses reveal that vascular networks were well integrated in the implanted muscle constructs (Kim and Kim, 2018). Choi et al. (2019) developed a hydrogel-based bioink utilizing decellularized extracellular matrix (dECM) for *granule-based coaxial nozzle printing* of muscle constructs (Figure 8). Pre-vascularization of the fabricated tissue with vascular *dECM bioinks* was found to prevent hypoxia as well as enhance muscle function recovery in rat models with volumetric muscle loss (VML). The *implanted pre-vascularized muscle tissue* exhibited improved *de novo* muscle formation in VML rat models (Choi et al., 2019).

Smooth muscles are anatomically and functionally different compared to skeletal muscle. Here, we describe one method of constructing vascularized smooth muscle tissue, specifically found in the urinary tract. Jin et al. (2020) used human adipose-derived stem cells in smooth muscle differentiation medium to grow *induced microtissues (ID-MTs)* which would be used as ink for the fabrication of urinary tract patches. VEGF and a tumor necrosis gene (TSG-6) were expressed in the ID-MTs for blood vessel growth (Jin et al., 2020). The *multilayered bioprinted structure* was transplanted into the subcutaneous tissue of nude mice. After just 1 week, vasculature was visualized in the functioning *3D printed smooth muscle construct* (Jin et al., 2020). Their results suggest that ID-MTs can be used along with vasculature-inducing factors for the development of blood vessels in fabricated smooth muscle tissue of the urinary tract (Jin et al., 2020).

#### Limitations and Future Suggestions

One of the most difficult portions of printing muscle using a printer is to make sure that the entire muscle fiber is printed and that each fiber will be printed and interacting with other fibers that will subsequently be printed. Along with printing the various types of muscle, some tissues such as the heart are a complex collection of various types of muscle. When it becomes possible to get muscle fibers printed then the next steps in the process will be to provide vascular connections so that a multilayer print can receive nutrients and eliminate waste, this is very important to also prevent ischemic injury to the tissues or the buildup of waste products such as lactic acid. After the completion of the very basic physiological needs of the muscle that has been printed the question arises if on this multilayer print there is also a need to go back in and make neuronal connections that will provide the muscle with the appropriate conductive signals to induce activation and also relaxation following neuronal activation. These different issues should be addressed over time and as there has been progress in printing muscle tissue and attempting to use VEGF to induce blood vessel formation 2 of the 3 key aspects for creating bioprinted replacements have been achieved. It remains to be seen once these printed muscles are implanted if the innervation naturally occurs.

### Vascularized Cardiac Tissues

Myocardial infarction and heart failure are the main causes of death in patients suffering from heart disease in the United States (Rodrigues et al., 2018; Wingard et al., 2020). Tissue engineering has offered promising alternatives when healthy cardiac tissue supplies from donors are lacking (Domenech et al., 2016). 3D printed constructs are viable options for patient implantation to ameliorate the life quality and enhance the prognosis of patients long term survival (Arai et al., 2018). One of the first *scaffold-free pumping cardiac tissues* was constructed using spheroids of induced pluripotent stem cells, endothelial cells, and fibroblasts as bioink printed on a needle array (Arai et al., 2018). Contractile movements and histological analysis of the construct mimicked that of biological cardiac tissue (Arai et al., 2018). However, this approach cannot produce full organs or large size tissues as no vasculature was included during printing (Arai et al., 2018).

**Figure 9:**
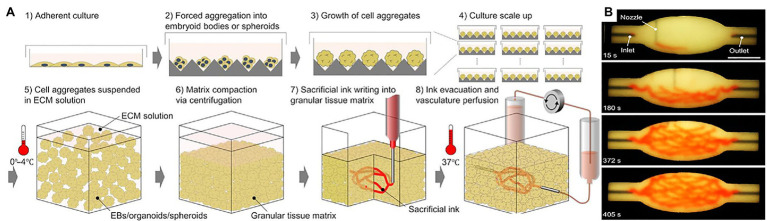
Bioprinting using organ building blocks. **(A)** Step-by-step illustration of bioprinting using organ building blocks (OBBs). **(B)** An image sequence showing the embedded 3D printing of a branched, hierarchical vascular network within a compacted EB-based tissue matrix connected to inlet and outlet tubes, seen entering the tissue from the left and right. Reprinted with permission from Sci. Adv. (Skylar-Scott et al., 2019), c 2021.

Larger viable cardiac tissue constructs require nutrient transport and waste disposal offered by imbedded blood vessel networks (Jafarkhani et al., 2019). In 2018, Maiullari et al. (2018) fabricated *cardiac tissue in vitro* with heterogeneous constructs made up of induced pluripotent cell-derived cardiomyocytes (iPSC-CMs) and HUVECs. They used an innovative approach of encapsulating the cells in hydrogel containing alginate and PEG-Fibrinogen (PF) and performing custom high-resolution spatial printing with a microfluidic printing head (MPH). The successfully *bioprinted cardiac tissue* product contained vessel-like networks that, through *in vivo* grafting, showed increased efficacy at supporting the integration of the fabricated product with the host’s vasculature (Maiullari et al., 2018). Skylar-Scott et al. (2019) focused on what they call *organ building blocks (OBBs)* consisting of patient-specific-induced pluripotent stem cell derived organoids to construct viable cardiac tissue (Figure 9). This rapid tissue assembly technique promotes self-healing behavior within damaged host tissue while maintaining high viscoplastic behavior (Skylar-Scott et al., 2019). They embedded *perfusable vascular channels via* 3D printing which resulted in a perfusable tissue capable of beating synchronously for 7 days while obtaining perfused nutrients through the 3D printed vascular channels (Skylar-Scott et al., 2019). These results show that this method of bioprinting cardiac tissue can highly mimic heart tissue behavior while maintaining compatibility with pre-printed vasculature (Skylar-Scott et al., 2019).

Previously, the fabrication of collagen scaffolds replicating the function and structure of cardiac tissue was a challenge. Lee et al. (2019a) successfully printed collagen constructs using *Freeform Reversible Embedding of Suspended Hydrogels (FRESH)* for vascular and cardiac tissue from capillaries to fully printed valves. The porous structure of collagen allowed rapid micro-vascularization of cardiac tissue while maintaining the mechanical strength of vasculature and tri-leaflet valves (Lee et al., 2019b). Although printing a fully functional organ is yet to be realized, this is a promising start with great potential toward the development of native cardiac tissues.

#### Limitations and Future Suggestions

The same limitations as discussed in the muscle section above are applicable here since cardiac tissue is a conglomeration of various muscle types and also have to include functional valves that can also be bioprinted. We will summarize some of the present limitations in creating a functional replacement for cardiac failure. As was outlined in the last section when attempts are made to print with various types of muscle to be incorporated into a viable print, the geometries are one of the first issues to get over and this is followed by assuring that all of the individual muscle types grow at comparable rates so that one type does not dominate as the attempt is to develop a replacement that functions in the same way as the native tissue.

To include vales in the print presents an additional set of problems in that the replacement vale needs to be biological and has the same location of intersection for the various leaflets that make up a valve. Presently, there has been some good progress to design valves that appear to have the same physiological properties as the original. If these were to be used, then they need to be affixed into the print which will require a vertical print where the complex muscles and vasculature are functional and then the valves need to be positioned in the lower portion of the print and then it would require that the second half of the chamber be developed above the seated valve. Due to the complexity of a multi-day multi-time print, it may be easier to print the two series of chambers and grow them in culture so that they remain viable while the valves are being printed. Once the various sections are assembled, it would be important to try some form of glue, either a surgical cyanoacrylic or a fibronectin-based glue. One issue with cyanoacrylic is that it typically gets hard and less flexible when exposed to higher levels of CO_2_ and or HCO_3_ which are abundant in tissues. Fibronectin based glues maybe a better alternative except the curing times are typically longer so that it may require multistage gluing; first, having valves attached to the distal part that would then be followed by having the proximal section of the print attached to the distal section containing the glued valves. An alternative idea is to use surgical sutures to attach the valves into position which can be done, as is commonly performed when porcine valves are placed in human tissues to replace a failing endogenous valve.

### Vascularized Liver Tissue

The liver is worth mentioning here because this organ is a critical hub for blood volume regulation and filtration (Trefts et al., 2017). Continuously filtering 100–130 ml/min per 100 g of tissue, the liver is intricately and highly vascularized (Lautt, 2009). There is a world-wide shortage of available transplantable livers, and among those fortunate enough to be matched, immune mediated injuries and the associated issues with immunosuppressive drugs remain an ever-present issue (Jadlowiec and Taner, 2016). 3D bioprinting with stem cells taken from the patient can potentially reduce host-rejection and eliminate the lifelong usage of immunosuppressants following a transplant (Tappa and Jammalamadaka, 2018).

Previously, incorporating vascular networks into thick and densely populated tissues, such as those found in the liver, was a challenge as traditional bioprinted tissue contained open vasculature with a square-lattice geometry, which results in reduced efficacy of direct perfusions (Pimentel et al., 2018). Therefore, vascular tissues also have to be thick and densely populated to reflect the needs of the target tissue (Pimentel et al., 2018). Pimentel et al. (2018) developed a method of printing *four arm branched vascular networks* with the same stiffness and density as that of the native liver. Results from this study showed that not only can the four arm branch networks be perfused to mimic physiological biological liver environments over extended periods of time (greater than 14 days; Pimentel et al., 2018). Liver cell densities and cell proliferation clustered around the artificial vascular networks (Pimentel et al., 2018). These findings reveal the significance of the incorporation of effective vasculature in thick and densely populated tissues such as the liver (Pimentel et al., 2018).

Recently, Yang et al. (2020) successfully printed *functional liver tissue* that exhibited normal liver functions such as ALBUMIN secretion, drug metabolism, and glycogen storage after 7 days of cell differentiation and growth in mouse models. Although no vascular designs were incorporated into the tissue constructs, the printing material used (hepatorganoids with the combination of HepaRG cells and bioink), has the potential to result in the development of functional vascular networks in transplanted tissues (Yang et al., 2020). The growth of the vasculature improved nutrient support and liver functions in the *3D printed construct*, ultimately leading to the prolonged survival of mice with liver failure (Yang et al., 2020).

#### Limitations and Future Suggestions

The issues to print a functional liver are similar to all other organs and tissues we have discussed in previous sections, there are multiple cell types that should be incorporated to allow for hepatic functions these are made of multiple special cells in the hepatocyte assemble process, along with conquering the multiple cell types, it is also essential to have a variety of vascular connections from capillaries to large arteries and veins. Cleary, one way to address the vascularization issue is what researchers in the above section have deployed, namely the use of VEGF to cause vessel formation from an endogenous source; however, this becomes more complex in the liver since there is such a large amount of native blood supply passing through the tissue in a given time that would mean that the VEGF may escape the tissue of interest and lead to potential tumorigenesis occurring at remote locations. Also, it will be important to address the large volumes of blood passing through the printed liver that could lead to leakage in and around the tissue that could lead to death.

### Vascularized Skin Tissue

Cost-efficient treatment of chronic, non-healing wounds is becoming increasingly important (Sen et al., 2009). In the United States, common types of chronic wounds include: venous ulcers, pressure ulcers, burn wounds, and diabetic wounds that affect over 7 million people each year with an annual cost of $25 billion (Sen et al., 2009). Split thickness autografts represent the present gold-standard of treatment, but they are limited by the availability of healthy donor skin (Jones et al., 2002). Allografts, on the other hand, are often associated with a strong inflammatory immune response against donor cells leading to a rejection of the graft (Morrow, 2004; Benichou et al., 2011). Dermal substitutes with or without cells can be costly to produce and have been shown to result in sub-optimal cosmetic outcomes (Albanna et al., 2019). Cellular therapy with epidermal keratinocytes and dermal fibroblasts derived from healthy skin utilize seeding or spraying methods that currently lack the high precision of delivery needed to generate complex skin structures (Albanna et al., 2019). In contrast, 3D bioprinting technology can deliver cells layer-by-layer to target sites with great accuracy (Murphy and Atala, 2014). The most commonly used type of printers for biological applications are inkjet printers in which droplets of solutions are dispensed onto a substrate from a cartridge that can contain a variety of materials, including cells (Cui et al., 2012; Yu et al., 2013).

**Figure 10:**
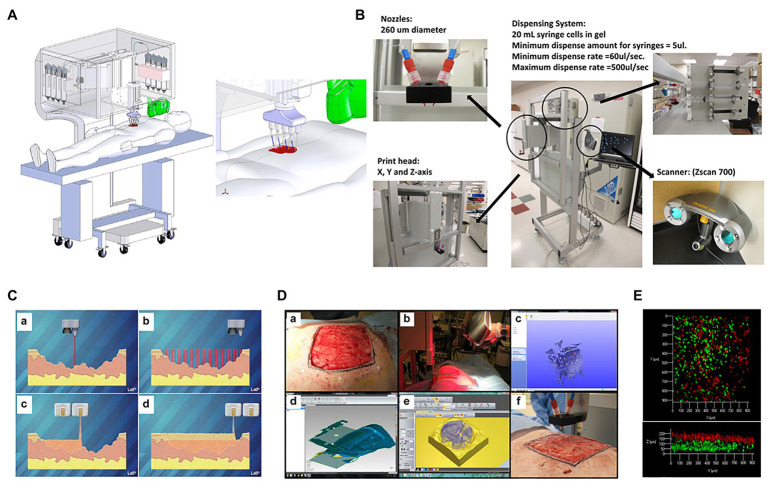
Skin bioprinter prototype and *in situ* bioprinting concept. **(A)** Schematic demonstrating scale, design, and components of the skin bioprinter. **(B)** The main components of the system consist of 260 μm diameter nozzles, driven by up to eight independently dispensing systems connected to a print-head with an XYZ movement system, in addition to the 3D wound scanner. All components are mounted on a frame small enough to be mobile in the operating room. **(C)** Skin bioprinting concept. Wounds are first scanned to obtain precise information on wound topography, which then guides the print-heads to deposit specified materials and cell types in appropriate locations (Images courtesy of LabTV – National Defense Education Program, Washington, DC, United States). **(D)** Example of skin bioprinting process, where markers that are placed around the wound area used as reference points **(a)** prior to scanning with a hand-held ZScanner™ Z700 scanner **(b)**. Geometric information obtained *via* scanning is then input in the form of an STL file to orient the scanned images to standard coordinate system **(c)**. The scanned data with its coordinate system is used to generate the fill volume and the path points for nozzle head to travel to print the fill volume **(d)**. Output code is then provided to the custom bioprinter control interface for generation of nozzle path needed to print fill volume **(e,f)**. Reprinted with permission from Sci. Rep. (Albanna et al., 2019), c 2021. **(E)** This system facilitates the depositing of multiple cell types with high precision and control. Layering of fibroblasts (green) and keratinocytes (red) is shown.

Recently, Albanna et al. (2019) conducted a proof-of-concept study of a *mobile in situ skin bioprinting system* with integrated imaging technology to provide rapid on-site management of full-thickness wounds (Figure 10; Albanna et al., 2019). Primary endpoints were defined as improved wound closure, re-epithelialization, and contraction, while a secondary endpoint was defined as healthy, mature skin formation as determined by histology (Albanna et al., 2019). In this study, they demonstrated the capabilities of their bioprinting system to deliver appropriate cell types and concentrations, by printing a *bilayered skin construct consisting of human fibroblasts and keratinocytes directly onto a full-thickness skin defect on a nude mouse model* (Albanna et al., 2019). Mice were divided into three groups of 12 animals each that consisted of no treatment, a printed group that received matrix only (solution of fibrinogen and collagen), and a cell-printed group that received a layer of human fibroblasts overlaid by another layer of keratinocytes (Albanna et al., 2019). All three groups of wounds were covered with triple antibiotic ointment followed by sterile gauze and surgical tape (Albanna et al., 2019). Evaluation of the wound area in mice over a 6-week period showed rapid closure of the wound in the cell-printed group compared to the control groups of bioprinted matrix and no treatment (Albanna et al., 2019). Overall, *printed skin cells* were able to close the entire wound by 3 weeks post-surgery compared to 5 weeks for both negative controls (Albanna et al., 2019). Subsequently, they tested the capabilities of the system by delivering *allogeneic or autologous dermal fibroblasts and epidermal keratinocytes within a biological hydrogel* to a large full-thickness wound in a porcine model and compared the results to bioprinted matrix only and untreated controls over 8 weeks (Albanna et al., 2019). The *in situ bioprinting of autologous cells* resulted in more rapid epithelialization and wound closure as well as reduction in wound contraction at the end of the study period compared to other groups. Pathologic examination correlated with gross appearance of the wounds and confirmed the viability of a bioprinted repair (Albanna et al., 2019).

One of the limitations in 3D bioprinting skin constructs is generating blood vessels in physiologically similar dimensions to the capillaries found in the skin microvasculature (Baltazar et al., 2020). The lowest diameter reported of a 3D bioprinted perfused vessel in a skin construct was 80 μm, compared to the physiologic measurement of <26 μm at the superficial horizontal plexus and <50 μm at the dermal-subcutaneous plexus (Braverman and Keh-Yen, 1981; Braverman and Sibley, 1990; Abaci et al., 2016). In a recent study, Baltazar et al. (2020) developed a work around for this limitation by *promoting vascular self-assembly in their bilayered skin constructs* that were morphologically and biologically similar to human skin. First, the vascularized dermal compartment was bioprinted with human foreskin dermal fibroblasts, human endothelial cells derived from cord blood human endothelial colony-forming cells, and human placental pericytes suspended in rat tail type I collagen (Baltazar et al., 2020). This layer was then cultured in Lonza EGM-2 endothelial cell growth medium for 4 days to promote vascular self-assembly. Second, the *epidermal compartment containing keratinocytes was bioprinted on day 4 and cultured* in skin differentiation medium. This 2-step approach allowed self-assembly of endothelial networks in the dermis as well as epidermal keratinization (Baltazar et al., 2020). In the clinical phase of their study, *bioprinted skin grafts without human endothelial cells or placental pericytes, with only endothelial cells, and with both endothelial cells and placental pericytes were implanted on the dorsal region of immunodeficient mice after 8 days of in vitro culture* (Baltazar et al., 2020). Bioprinted grafts without endothelial cells and placental pericytes were significantly smaller with a larger area occupied by endogenous mouse skin. *Grafts with human endothelial cells contained vascular structures 4 weeks post-engraftment* (Baltazar et al., 2020). The presence of placental pericytes in the printed dermis not only improved keratinocytes maturation and formation of epidermal rete, but also enhanced development of host microvessels into the graft, thereby allowing perfusion through both graft and host microvessels (Baltazar et al., 2020). This exciting observation shows the potential of incorporating this technology for clinical applications.

In this final section of the review, we will discuss another interesting application of *3D Bioprinting*, namely the development of synthetic organoid platforms that will act as *in vitro* mimics of various organ systems to allow for the examination of new targeted pharmaceutical and chemolytic agents.

#### Limitations and Future Suggestions

The development of bioprinted skin is an exciting idea and a potential way to treat many abnormalities that can occur across the skin such as but not limited to: cancer, trauma, both cuts and burns, abnormalities due to radiation injury for other disease treatments, and deterioration of the blood supply leading to necrosis as can occur with diabetes. The skin also needs a blood supply and that this would be the first area to address once the keratinocytes have been printed. These can come from harvested dissociated skin, or potentially they may be replaced with stem cells from the individual. With these various techniques being deployed, the need for a blood supply becomes the most difficult to maintain the bioprinted implant. Researchers are again reaching out to use VEGF to stimulate vessel formation but the same issue as described above remains to keep the VEGF localized. Another issue is the high turnover in skin of cells so that implanted skin will need to grow and sluff off at the same rates as the native tissues to prevent additional abnormalities that would be detrimental to the recipient.

### *In vitro* Models for Drug Assays

Presently, many *in vitro* developed models have been employed to screen medications and to examine healthy and diseased states in a controlled environment that attempts to mimic the normal physiology of the tissue or organ (Pennarossa et al., 2021). The ability to test agents in both normal and diseased cells can lead to improved screening of agents and discoveries for improved long term outcomes for the patient. In the following sections, we will explore some of the current advances in *in vitro* models of tissues with more complex architectures and organization for drug screening and assays.

#### Limitations and Future Suggestions

The use of “organoids” is a growing field as a means for performing toxicity testing on a variety of tissues as new agents come along. This has been used for chemolytics along with other blood pressure agents to date. The potential advantage of these organoids is that you can rapidly screen agents on human derived tissues so that you can eliminate the need for animal testing. The limitation is are these small clusters of cells behaving in the same way as the native tissue that they were derived from or have they been modified by the culture conditions to grow them. We have addressed the use of cells and the associated vasculature and also nervous tissue so that they may need to make larger organoids that incorporate not only the cells of interest but a blood supply and neuronal intervention to determine if the organoid is truly mimicking the native tissue. Furthermore, the tissues are going to have to be artificially perfused so that the mediate will need to be oxygenated and exchanged on a regular basis to prevent the buildup of waste materials.

### Vascularized Intestinal Villi

**Figure 11:**
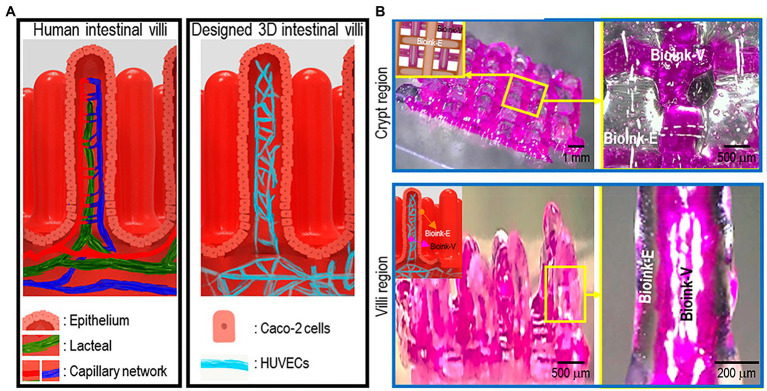
The 3D intestinal model. **(A)** the designed 3D intestinal model. **(B)** Optical (transparent, epithelium; and pink, capillary) images of the crypt and villus regions for the fabricated intestinal model. Reprinted with permission from ACS Appl. Mater. Interfaces (Han et al., 2020), c 2021.

Kim and Kim (2018) successfully 3D printed the intestinal models containing villi structures and capillary structures with a dual-cell-printing process (Figure 11). Two collagen-based bioinks were developed using Caco-2 cells in one and HUVECs in the other. Not only did the 3D structure mimic the function of actual intestinal villi, as confirmed by increased permeability to glucose (Kim and Kim, 2018). Cellular activity of the intestine models with capillary structures had increased cell growth and expression of digestive enzymes and MUC17, a membrane-bound mucin that protects gut epithelial cells (Kim and Kim, 2018). This fabricated vascularized intestinal model is a promising start to the construction of fully functional human intestines (Kim and Kim, 2018).

#### Limitations and Future Suggestions

The idea of creating vascularized intestinal villi is an exciting one since this would help to address a large clinical need where to date the level of transplantation has been small. In some recent work from our group, we have been able to demonstrate *in vivo* that a patch created from a combination of smooth muscle and fibroblasts can incorporate into the native tissue and the animal can survive and grow at the same rate as sham controls. What was exciting in this study was that we could show that the endothelium containing crypts and villi migrate across the patch and develop a normal lining in approximately 7 days. For the future the idea to implant an entire section of bioprinted tubular material is exciting. The limitation may be that if the implant is long, it may not be able to reestablish the endogenous lining from the proximal and distal sites of anastomosis. Along with this issue is to make sure that a longer section could be attached to the blood supply rather than wait for the supply to develop, the length will also need to be innervated so that peristalsis can continue across the implant and prevent the development of pools of waste in the print that may lead to a blockage. The next steps in addressing this process are to make longer and longer patches to determine if there is an upper limit as to length for normal endothelial development across the patch. Once these limits have been established, then full tubular replacements can be attempted.

### Vascularized Retina Tissue

The mammalian retina consists of multiple interconnected cellular layers that require a continuous and abundant blood supply both anteriorly and inferiorly (De Moraes, 2013). Recently, Masaeli et al. (2020) bioprinted vascularized retina tissue for *in vitro* drug testing. Using scaffold-free techniques and carrier-free inkjet bioprinting, a layer of photoreceptors was deposited onto a printed layer of retinal pigment epithelium. The release of VEGF from the retinal pigment epithelium layer resulted in the formation of vascular networks for the optimized functioning of the printed layers (Masaeli et al., 2020). Controlling the placement VEGF release during the construction of the tissue or organoid offers a method of designing and modeling vascular architecture similar to that of the native tissue counterpart. However, due to the carcinogenic nature of VEGF, prints utilizing this method need to be thoroughly investigated in *in vivo* models before any human implantation (Verheul and Pinedo, 2000; French and Frazier, 2011). Although the construct lacks the complexity to that of biological human retina for surgical implantation, the print products could be used to study loss of vision diseases such as retinitis pigmentosa and age-related macular degeneration to observe cell behavior and tissue development upon disease onset (Masaeli et al., 2020).

#### Limitations and Future Suggestions

The present state of implantation in the retina is that cellular constructs can be printed and research is underway to explore using VEGF to vascularize the print. As discussed above, the researchers in this field are aware of the issues with using VEGF due to its support for tumor growth so that alternative vascular growth agents need to be investigated that may not have these associated issues. It appears that printing extremely small capillary beds will not be possible with the present print technologies so the deployment of these prints will require additional work. One thing that should also be remembered is that VEGF should be applied in the prints and then look at histology to determine if VEGF also modified the printed cells in an adverse way. If this appears to be the case, then extreme caution is needed before moving on to implanting in an animal model system.

### Kidney Proximal Tubule Models

Proper vasculature is essential for the functions of the kidneys for water, electrolyte, and nutrient filtration and absorption back into the bloodstream (Lin et al., 2019). Lin et al. (2019) produced functional vascularized kidney and proximal tubule models by printing adjacent conduits lined with confluent endothelium and epithelium embedded in a permeable extracellular matrix. The tubule epithelium and vascular endothelium construct displayed similar reabsorption properties to that of native kidney tissue (Lin et al., 2019). Diseased kidney states can be modeled by exposing the construct to hyperglycemic conditions. This model could be used to study specific aspects of kidney function (i.e., proximal tubule vs. thick ascending and descending limb or collecting duct) and disease modeling *in vitro* (Lin et al., 2019). It is important to point out that the complex pathophysiologic aspects of fluid and electrolyte movement and hormonal interactions, as well as bioprinting the full architecture of the kidney and renal tubule, remain an elusive target due to the wide range of cell specialization and function within the kidney. This is compounded with the need to create a printed tissue with millions of nephrons similar to a normal kidney that are highly vascularized and contain many different cell types and a large variety of intraluminal diameters. In addition, the collecting duct has many branches to allow different nephrons to empty into this central conduit (Seldin and Giebisch, 2008).

#### Limitations and Future Suggestions

In the previous section, we discussed some of the issues associate with attempting to print the proximal tubule in the kidney. It should be pointed out that the kidney has three different segments in the proximal tubule and that the length and diameter are slightly different in the cortex nephrons and in the medullar nephrons. All nephrons are encased with a vascular network that allows for the secretion and absorption of nutrients and elimination of waste products. To address the issue of providing a blood supply, the first efforts should again involve the use of VEGF to try and establish a vasculature for the proximal tubule. Should this work then becomes the next larger issue in that a kidney has approximately 2 million nephrons so that would require massive efforts to print just the 2 million proximal tubules per kidney, and these alone would not have use unless deployed in a culture model for drug toxicity. This would require a perfusion device to enter the lumen of the nephron and determine function. What we did not address is that the apical surface of the proximal tubule has a variety of villi on the apical surface and the height of the villi changes in the S1, S2, and S3 segments of the proximal tubule. This leads to additional issue that will make a viable proximal tubule to require a great deal of additional research for any use.

### Tumor Modeling

**Figure 12:**
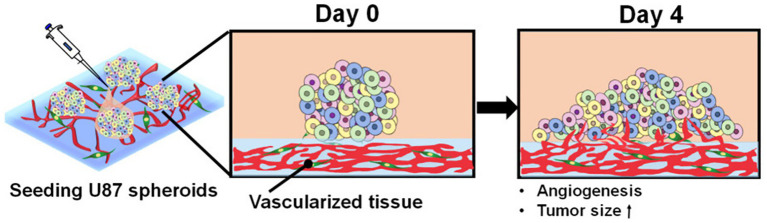
Angiogenesis of tumor spheroids. Schematic describing the morphological changes and angiogenesis of tumor spheroids of glioblastoma cells seeded onto the vascularized tissue. Reprinted with permission from Int. J. Mol. Sci. (Han et al. 2020), c 2021.

Out of all types of cancers, glioblastoma, the most diagnosed primary malignant brain tumor, is known for its extensive and abnormal growth of vascular networks (Wang et al., 2020a). However, the mechanism of tumor vascularization and angiogenesis is still controversial (Wang et al., 2020a). Wang et al. (2020b) developed a 3D printed hydrogel scaffold for the construction of the glioblastoma microenvironment *in vitro* with two glioblastoma cell lines, U118 and GSC23. Although both U118 and GSC23 cell lines exhibited good printability, GSC23 had an increased ability of secreting VEGF and forming vascular-like structures (Wang et al., 2020a). This new *in vitro* model for vascularized glioma tissue utilizing the GSC23 cell line could potentially be used for research in glioma cell behavior, glioma vascularization, and targeting of angiogenesis in tumors (Wang et al., 2020a). Han et al. (2020) constructed vascularized glioblastoma models for *in vitro* drug testing with an emphasis on the tumor microenvironment. The tumor microenvironment was created by printing a blood vessel layer using fibroblasts and endothelial cells followed by seeding tumor spheroids of glioblastoma cells onto the blood vessel layer (Figure 12; Han et al., 2020). The increasingly growing tumor spheroids induced angiogenesis from the blood vessel layer as expected for cells in a tumor microenvironment (Han et al., 2020). The treatment with the anti-cancer drug temozolomide and angiogenic inhibitor sunitinib resulted in similar inhibition of tumor and vascular growth as that of real cancer tissues (Han et al., 2020). These results suggest that the bioprinted tumor microenvironments can be an effective *in vitro* testing platform for vascularized tumors (Han et al., 2020).

#### Limitations and Future Suggestions

The development of tumor models with bioprinting is an area of exceptional promise. If the cells can be isolated from the tumor, they could be grown in culture and then harvested for printing a 3D model of the tumor *in situ*, this then gives an important tool for targeted drug delivery to destroy the tumor and since the tumor was grown from cells from the patient it would allow for further tailor-made designed therapies that should have high selectivity for the tumors and prevent future growth or recurrence. The future suggestion for these studies is to deploy a similar strategy that is presently being deployed for organoids as outlined in the last section of this proposal.

### Vascularized Tissue on Chips

Organ-on-chips, or tissue chips, are 3D platforms designed and engineered to support living tissues and cells (Miri et al., 2019). Once developed, these devices can be used to test the efficacy of candidate drugs, vaccines, or biological agents on specific cell and tissue types (Yi et al., 2017; Miri et al., 2019). Organ-on-chips can be incorporated into high throughput assays to allow faster and more cost-effective testing methods (Miri et al., 2019). 3D printing techniques are now being utilized in tandem with organ-on-chips for faster and cheaper productions of organ like cellular conglomerates (Yi et al., 2017). However, the success of this technology relies on how accurate the printed tissue can mimic that found in hosts. Thus, vascularization in fabricated tissues remains an essential component for mimicking function and nutrient exchange in drug testing and assays.

**Figure 13:**
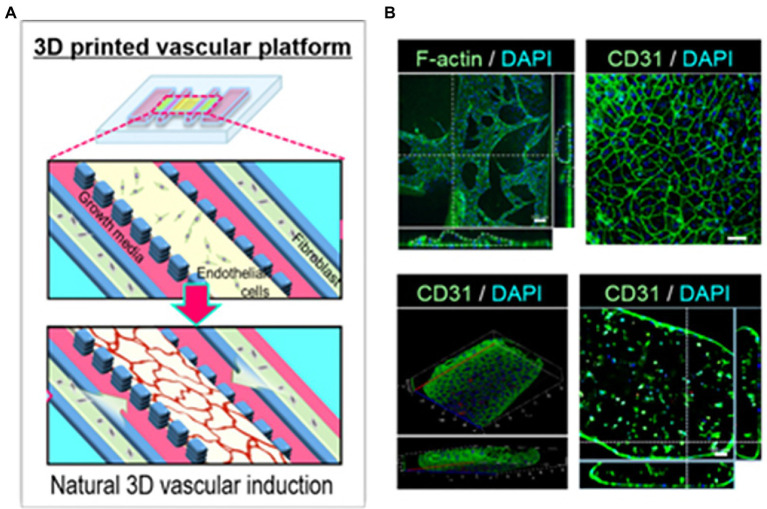
Vascular network formation on the 3D cell printed vascular platform (VP). **(A)** Design of VP for induction of natural 3D vascular network. **(B)** Confocal microscope images of *z*-stacked 3D vascular network formed on VP (top panel, left, scale bar: 100 μm) and EC junctions formed on the surface of vascular network (top panel, right, scale bar: 50 μm). Confocal microscopy images of vascular tube formation by *z*-stacked (bottom left) and transverse cross-sectional (bottom right, scale bar: 50 μm) views. Reprinted with permission from Biofabrication (Park et al., 2018), c 2021.

Park et al. (2018) constructed an *airway-on-a-chip* mimicking respiratory tissue by printing endothelial cells and fibroblasts encapsulated in porcine tracheal mucosa derived extracellular matrix into naturally derived vascular networks *in vitro* (Figure 13A). By mimicking *in vivo* conditions, a functional lumen and blood vessel network formed providing an interface between the airway epithelium and the blood vessel network (Figure 13B; Park et al., 2018). This vascularized tissue-on-a-chip model was able to exhibit respiratory symptoms such as asthmatic inflammation and allergen-induced asthma exacerbation under the right induced physiological conditions (Park et al., 2018). The high similarities between the *in vitro* model and the corresponding biological tissue as well as the high reproducibility due to the high specificity of 3D printing makes these high-content platforms good candidates for preclinical trials in drug research (Park et al., 2018).

**Figure 14:**
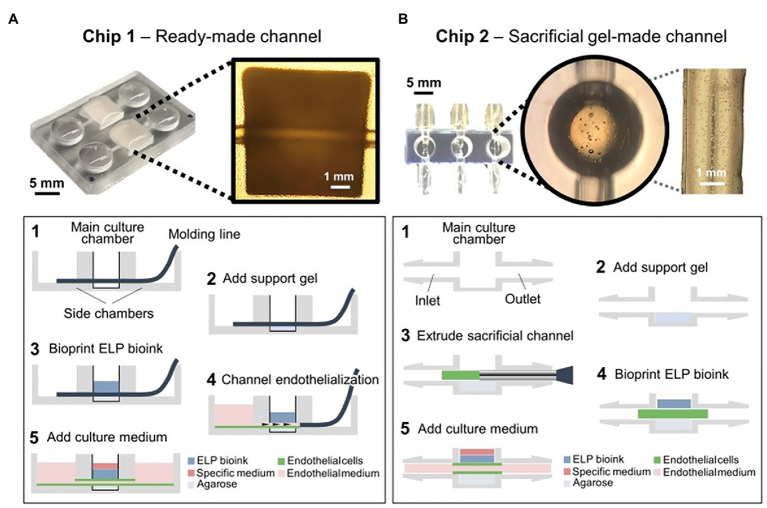
Chip designs used for the biofabrication of printed tissue frameworks with on-chip vascular-like channels. Chips designs with **(A)** ready-made channels and **(B)** sacrificial gel-made channels were evaluated. Sequence of events (1–5) indicates the necessary working steps to fabricate both types of tissue models. Sacrificial gel-made channels can be used to incorporate endothelial cells that will form monolayers on the channel surface after removing the sacrificial gel. The inset photograph in **(A)** shows the channel formation at step 5. The inset photograph in **(B)** shows the presence of endothelial cells inside the sacrificial gel at step 3. Reprinted with permission from Front. Bioeng. Biotechnol. (Duarte Campos et al., 2020a), c 2021.

So far, 3D bioprinting technology could only print relatively simple structures of vascularized tissue with low heterogeneity (Duarte Campos et al., 2020b). Duarte Campos et al. (2020a) in a proof-of-concept study, directly printed neural progenitor cells and breast cancer spheroids encapsulated in elastin-like protein engineered hydrogel bioinks onto endothelialized on-chip platforms (Figure 14). Cells remained viable after 14 days with enduring vascular-like channels suggesting the successful development of a functional tissue on a chip (Duarte Campos et al., 2020a). These are the first steps toward establishing fully functional heterogeneous tissue models with vascularization on-chip platforms for *in vitro* biomedical applications and testing (Duarte Campos et al., 2020a).

**Figure 15:**
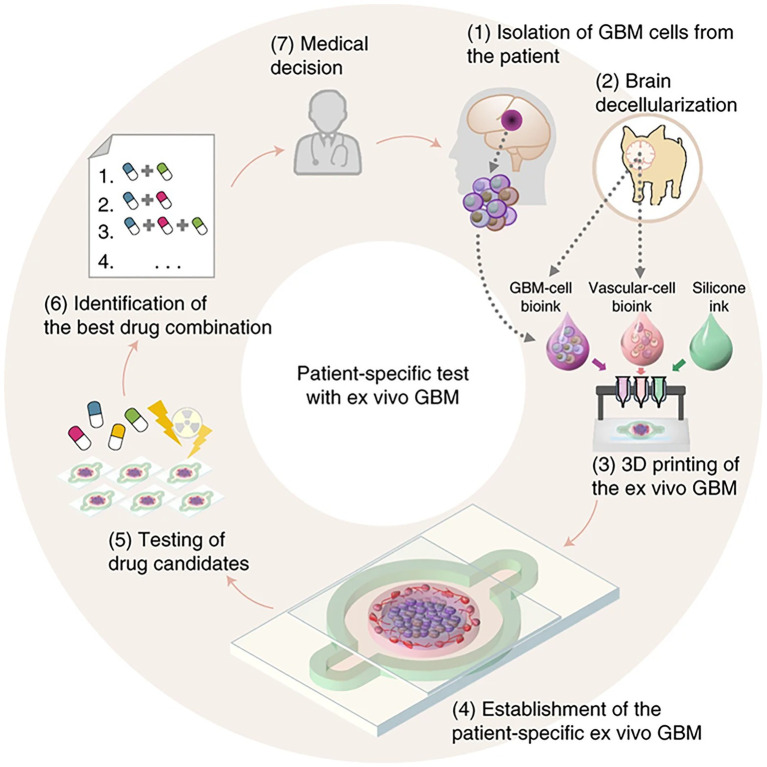
Flowchart for building tissue-chip-models for glioblastoma. Step 1, GBM cells are isolated from a specimen obtained through removal surgery. Step 2, off-the-shelf porcine BdECM bioink is obtained. Step 3, patient-derived cancer cells are printed with the BdECM bioink to produce a patient-specific GBM-on-a-chip. To mimic the heterogeneous GBM ecology, several other inks are used in the printing process, including a vascular cell-laden BdECM bioink and a silicone ink. Step 4, the chip is cultured for 1–2weeks to recapitulate the pathological features. Step 5, various candidate drug combinations are tested using the chip. Step 6, the drug combinations are prioritized according to their efficiencies and the best combination is identified. Step 7, the physician uses the test results to design a treatment plan for the patient. Reprinted with permission from Nat. Biomed. Eng. (Yi et al., 2019), c 2021.

Recently, Yi et al. (2019) developed *patient specific tissue-on-a-chip models for glioblastoma* (Figure 15). Using native patient-derived tumor cells, and vascular endothelial cells and decellularized extracellular matrix for porcine brain tissue, reconstituted glioblastoma tissues were printed in a compartmentalized cancer-stroma concentric-ring structure for sustained radial oxygen gradients (Yi et al., 2019). This *in vitro* model exhibited patient-specific resistances to anti-cancer treatments of chemoradiation and temozolomide. This technique of printing patient specific diseased tissue can be utilized for determining effective drug combinations for patients resistant to standard first-line treatments (Yi et al., 2019).

#### Limitations and Future Suggestions

The idea of vascularized tissues on a chip is intriguing and presents a unique and novel way to look at disease states and how to potentially design reagents and therapies that can later be applied to patient health. Incorporating vasculature into a tumor is important for drug design and also to determine if you target the tumor or just the associated vasculature or both. It also becomes an ideal target for genetic therapy as it can be examined directly prior to moving to healthy tissues. This is one of the limitations that comes up since it will become critically important to also develop a printed model of the associated healthy tissues to test the same therapies on to assure that the planned therapy will have minimal side effects in the patient that the therapy was designed for.

## Conclusion

The incorporation of vascular networks in engineered tissue constructs, while maintaining tissue integrity, remains a challenge that needs to be addressed before any human implantation applications can happen (Lovett et al., 2009; Sarker et al., 2018). 3D bioprinting offers promising advancements in this field of research by offering its ability to construct complex multi-material tissue architecture with a high degree of resolution. Current research has led to initial success in developing tissue(s) *in vitro* with embedded tubular vessel constructs for drug and pathogen testing (Satpathy et al., 2018). As of now, the most seemingly feasible method of host implantation involves the incorporation of VEGF in bioinks to induce host vascular growth into the implanted tissue (Sarker et al., 2019). Although promising, potential dangers of utilizing growth factors involve increased risk of tumorigenesis and cancer development (Witsch et al., 2010). There has been a large variety of printing methods, bioinks, and scaffolds used in constructing just one specific tissue type. However, comparative studies on the efficacies of the different constructs in terms of vascular nutrient transport and tissue viability are lacking (Chen and Liu, 2016). There have been many studies performed on animal models and cell lines, but they often fail to emulate human tissue behavior and contribute to difficulties in translating to human clinical trials (Jovic et al., 2020). Although current 3D printed vascular network scaffolds are premature for human implantation, simplified tubular blood vessel constructs are adequate for the use of drug screening and high throughput assays (Yu and Choudhury, 2019). More research is necessary for the development of constructs mimicking complex tissue types such as the human retina and the kidney. Furthermore, there is no suitable tissue culture device to simulate and evaluate the degree to which fabricated tissues mimic its biological counterparts (Yu and Choudhury, 2019).

With the current advances in the incorporation of vasculature into 3D bioprinted tissue and organs, bioprinting remains one of the most promising methods to construct vascularized tissue for implantation that mimics the native tissue that it will replace or support. However, research still needs to be done before 3D printed vascularized tissue constructs can be widely used in human patients (Dimitrievska and Niklason, 2018; Pennarossa et al., 2021). Meanwhile, the use of fabricated vascularized tissue for *in vitro* applications such as drug screening and high throughput assays in clinical trials is feasible and would most likely be employed in the near future, thereby reducing the costs for drug development while providing important preclinical information that could be used for both toxicity and efficacy of targeted action of the agent.

## Author Contributions

JG proposed the topic and supervised and edited the manuscript. EC and ZT researched the topic and wrote and edited the manuscript. BD edited and wrote a portion of the manuscript. All authors contributed to the article and approved the submitted version.

### Conflict of Interest

The authors declare that the research was conducted in the absence of any commercial or financial relationships that could be construed as a potential conflict of interest.
